# Metabolite Profiling of Leaves of Irises and Cinquefoils of Hydrophytic and Mesophytic Nature

**DOI:** 10.3390/ijms27041814

**Published:** 2026-02-13

**Authors:** Sergey A. Vanisov, Pavel D. Smirnov, Roman K. Puzanskiy, Oleg S. Butorlin, Alexey L. Shavarda, Maria F. Shishova, Vladislav V. Yemelyanov

**Affiliations:** 1 Department of Plant Physiology and Biochemistry, Faculty of Biology, St. Petersburg State University, Universitetskaya Embankment, 7-9, 199034 Saint Petersburg, Russia; s.vanisov@mail.ru (S.A.V.); mshishova@mail.ru (M.F.S.); 2 Department of Botany, Faculty of Biology, St. Petersburg State University, Universitetskaya Embankment, 7-9, 199034 Saint Petersburg, Russia; 3 Department of Genetics and Biotechnology, Faculty of Biology, St. Petersburg State University, Universitetskaya Embankment, 7-9, 199034 Saint Petersburg, Russia; puzansky@yandex.ru; 4 Laboratory of Analytical Phytochemistry, V.L. Komarov Botanical Institute of the Russian Academy of Sciences, Professora Popova Str., 2, 197376 Saint Petersburg, Russia; 5 Institution of Chemistry, St. Petersburg State University, Universitetskiy Prospect, 26, 198504 Saint Petersburg, Russia; 6 Center for Molecular and Cell Technologies, Research Park of St. Petersburg State University, 199034 Saint Petersburg, Russia

**Keywords:** hypoxia, hydrophyte, mesophyte, metabolomics, GC-MS, *Iris pseudacorus*, *I. sibirica*, *Comarum palustre*, *Argentina anserina*

## Abstract

Plants living in wetland environments (hydrophytes) have developed metabolic adaptations to cope with hypoxia. However, the specific metabolic signatures underlying this adaptation in naturally occurring hydrophytes, compared to their mesophytic relatives, remain insufficiently explored. GC-MS was used to carry out a comparative metabolic profiling of leaves from two pairs of closely related species (the hydrophytes *Iris pseudacorus* and *Comarum palustre* versus the mesophytes *I. sibirica* and *Argentina anserina*). In total, approximately 260 compounds were detected, of which roughly 100 were annotated. Using Principal Component Analysis, we revealed that the primary source of metabolic variation was phylogenetic (genus/tribe affiliation), while a secondary gradient correlated with ecological adaptation to submergence. A common adaptive metabolic signature of hydrophytes was identified, including the accumulation of dicarboxylic acids from Krebs cycle (succinate, fumarate) and glycolytic metabolites (pyruvate), suggesting a restructuring of energy metabolism under oxygen deficiency. Furthermore, hydrophytes, particularly *I. pseudacorus*, accumulated higher levels of soluble sugars (sucrose, fructose, glucose) and amino acids, thereby supporting energy supply and alternative NAD(P)H reoxidation pathways. Species-specific differences in the accumulation of antioxidants (e.g., flavonoids, ascorbate) were also observed, indicating diverse strategies for managing oxidative stress. Our findings contribute to identifying a “metabolic fingerprint” associated with waterlogging tolerance.

## 1. Introduction

Plants, as sessile organisms, have evolved intricate metabolic networks to adapt to diverse environmental challenges, ranging from abiotic stressors like drought and salinity to biotic interactions with pathogens and herbivores. Among these challenges, adaptation to aquatic and semiaquatic environments—a niche occupied by hydrophytic species—presents unique physiological and biochemical constraints, including hypoxia, fluctuating nutrient availability, and oxidative stress. While significant progress has been made in understanding responses of plant metabolism to drought and extreme temperatures, the metabolic strategies enabling survival in hydrophytic habitats remain comparatively underexplored [1,2]. This knowledge gap is particularly critical as climate change alters hydrological regimes, necessitating a deeper understanding of plant resilience mechanisms in wetland ecosystems.

Metabolic profiling is a powerful tool that allows us to investigate changes at the molecular level. It involves the identification and quantification of the entire set of metabolites in biological samples, which provides insight into the metabolic adaptations of plants to different environmental conditions. Metabolomics allows us not only to identify the metabolites involved in adaptation to oxygen deprivation, but also to monitor changes in their abundance in response to various environmental stimuli.

Hypoxia (partial oxygen deficiency) and anoxia (complete absence of oxygen) are common in nature, especially in cases of waterlogged soils, which is typical for floods or seasonal high waters. These conditions are critical for winter crops (wheat, barley), rice, soybeans, cotton, and some trees (willow, spruce) [3]. During hypoxia, mitochondrial ATP production falls below the level required to supply cells with energy: the main ways of obtaining energy are blocked—photosynthesis and aerobic respiration [4]. Hypoxia slows down the synthesis of phospholipids and increases lipid peroxidation. In resistant species, the unsaturation index of fatty acids in mitochondria and chloroplasts is preserved for longer, which maintains membrane fluidity and functional activity [3]. Oxygen deprivation suppresses protein synthesis due to degradation of polysomes and mRNA [3]. Hypoxia itself and subsequent reoxygenation increases the production of reactive nitrogen and oxygen species, causing oxidative damage [5,6,7].

Resistance to hypoxia or mitigation of its negative impact can be achieved in plants in various ways. There are two strategies of plant adaptation to hypoxia. First, plants try to avoid the impact of the unfavorable factor (low oxygen escape syndrome, LOES): flooded parts of plants actively elongate axial organs—processes occur to quickly restore contact of oxygen with tissues [3,8]. A superficial adventitious root system develops, special air-bearing porous tissue, aerenchyma, appears in the plant body, gas exchange occurs through lenticels and stomata, and oxygen is transported to the roots [3,9]. Second, plants can restructure their metabolism for long-term survival in hypoxic conditions or for a full life in such conditions (low oxygen quiescence syndrome, LOQS) [8,9,10]. These changes include: a decrease in the overall intensity of metabolic processes, redistribution of limited resources of macroergic compounds to support vital functions, maintain the integrity and activity of membranes, the synthesis of stress proteins, as well as pH regulation to prevent the acidosis of the cytoplasm [3,10]. Both strategies rely on a metabolic background that allows tolerant plants to produce sufficient energy, maintain water intake, and even grow under oxygen-deficient conditions (for LOES plants).

Studies of adaptation to hypoxia are mainly carried out with the representatives of cultivated plants or with the model objects. The metabolic profile of plants growing in a flooded environment differs significantly in primary metabolites from those that live on soils with normal moisture. Oxygen deficiency activates glycolysis and fermentation in the majority of plants, resulting in the accumulation of lactate, acetaldehyde and ethanol; acidification of the cytoplasm is also observed [3,8,9,11]. Among the Krebs cycle metabolites, the abundance of succinate and fumarate is increased, while the levels of other components such as citrate and malate are decreased. Accumulation of succinate results from the reversal of the dicarboxylic part of the Krebs cycle, leading to the transformation of oxaloacetate into malate, or stimulation of GABA shunt [2,3,12]. It is not associated with the glyoxylate cycle, since isocitrate lyase is suppressed by oxygen deficiency in plant tissues, regardless of their resistance to such a condition [3]. There is an accumulation of amino acids associated with glycolysis (Ala, Gly) and the Glu metabolism (GABA, Pro, Arg), which helps to provide the alternative regeneration of oxidized NAD(P)^+^, maintain osmotic adjustments, and prevent lactoacidosis and accumulation of toxic fermentation products [2]. Despite the fact that different analytical platforms (GC-MS, LC-MS, CE-MS and NMR), varied intensities (hypoxia or anoxia), and different durations of oxygen-depleted exposure were used in the diverse experiments, various details of the metabolic profiles have been shown in different plants like Arabidopsis [13], *Lotus japonicus* [14], soybean (*Glycine max*) [15,16], rice [17,18,19], wheat (*Triticum aestivum*) [20], and barley (*Hordeum vulgare*) [21]. Nonetheless, plants that are more tolerant to hypoxia (rice, Arabidopsis) seem to accumulate higher amounts of amino acids (Ala, Glu and GABA) and succinate [2]. Metabolic profiling of wetland species like pondweed (*Potamogeton anguillanus*) [22] and eelgrass *Zostera marina* [23], hydrophytic willow-herbs (*Epilobium* spp.) [24] and buttercups (*Ranunculus* spp.) [25] revealed the same tendencies. Moreover the limitations in the data set that was available for sensitive plants does not allow a conclusion to be made on specific alterations in the metabolic processes.

The comparative metabolic profiling of closely related hydrophytic and mesophytic plant species represents a highly promising approach for studying plant adaptation to hypoxia. The members of genus *Iris* (Iridaceae) and cinquefoils (tribe Potentilleae (Rosaceae)) represent two ecologically and phylogenetically distinct lineages that have independently colonized both mesophytic (moderately moist) and hydrophytic (water-saturated) habitats. *Iris sibirica* and *I. pseudacorus*, for instance, exhibit contrasting ecological preferences: the former thrives in mesic grasslands, while the latter dominates wetlands and shallow waters [26]. Similarly, within Potentilleae, *Comarum palustre* (marsh cinquefoil) is a hydrophyte, specializing in waterlogged environments such as peat bogs and fens, where it demonstrates distinct adaptations to saturated soils and even periodic flooding [27]. In contrast, *Argentina anserina* (=*Potentilla anserina*, silverweed or silver cinquefoil) exhibits the broader ecological amplitude of a mesophyte, living in moderately moist to dry, disturbed habitats like roadsides and overgrazed meadows, where its competitive success is linked to its tolerance to disturbance rather than waterlogging [28]. These genera thus offer a persuasive comparative framework to explore how phylogeny and ecology intersect to shape metabolic phenotypes.

Data is available on the metabolic profiling of representatives of the genus *Iris* and the cinquefoils focused on the investigation of biologically active compounds for pharmacological and therapeutic use [29,30,31,32]. Our study aimed to compare the metabolic profiles of the leaves of two irises and two cinquefoil species from the tribe Potentilleae of hydrophytic and mesophytic nature to identify characteristic patterns of the metabolome. It has to be mentioned that representatives of the tested species were collected from native habitats that differed in available humidity. The obtained results are of interest for plant ecological metabolomics.

## 2. Results

### 2.1. Metabolite Profiling

**Figure 1:**
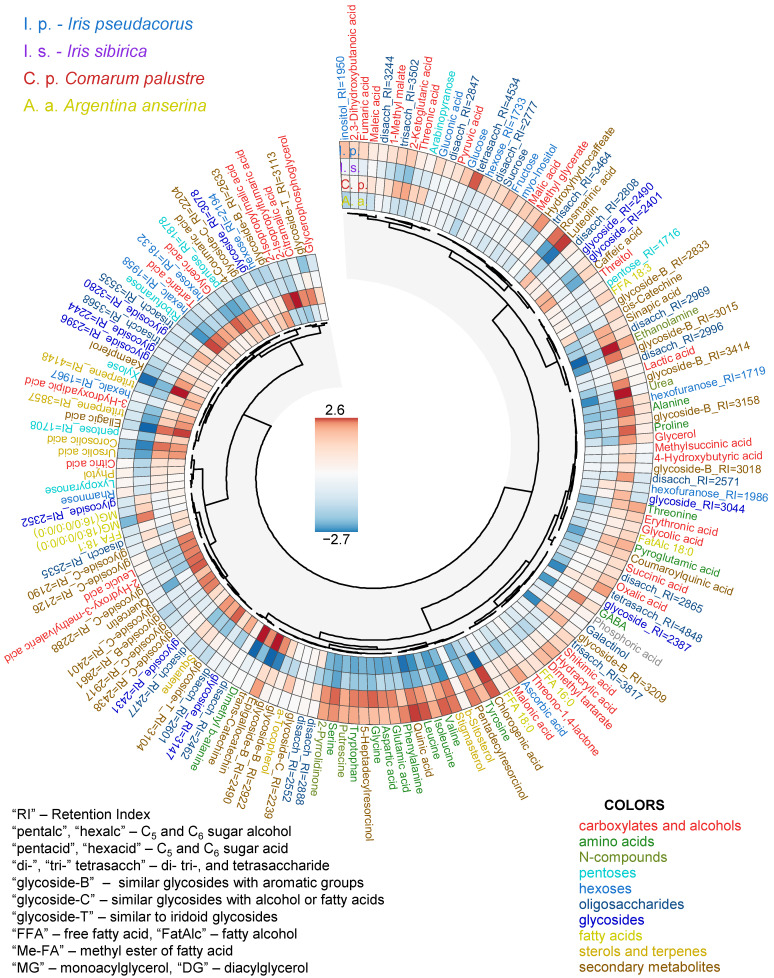
Heatmap of normalized arbitrary metabolite content. Metabolites are clustered by Pearson distances (1-*r*) by Ward method.

**Figure 2:**
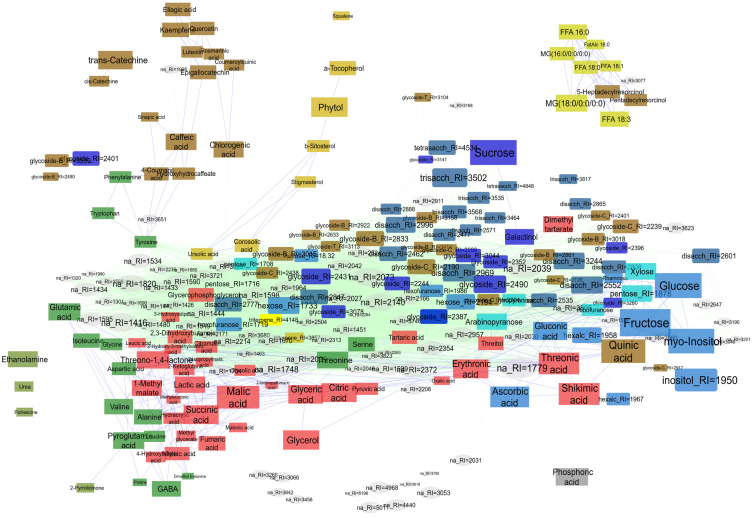
Metabolites molecular mapping. Blue edges correspond to similarity of identified molecules (Tanimoto index > 0.3) and green edges to mass-spectra similarity (cosine similarity > 0.8) for unidentified components. Edges constrain nodes, node colors correspond to compound classes, and sizes reflect median arbitrarily normalized content.

Using gas chromatography-mass spectrometry (GC-MS), we conducted metabolite profiling of leaves from two species of the genus *Iris* (*I. pseudacorus* and *I. sibirica*) and two cinquefoil species (*C. palustre* and *A. anserina*). A total of approximately 260 compounds were detected. About 100 metabolites were annotated using spectral databases, while an additional 65 compounds were classified into broader metabolite categories (Figure 1 and Figure 2). The profiles were predominantly characterized by sugars and their derivatives, including pentoses (7), hexoses (14), oligosaccharides (22) and over 60 glycosides. Precise identification of glycosides was challenging due to their structural diversity and the similarity of mass spectra produced by sugar moieties. Based on spectral similarity to library entries, we categorized oligosaccharides (di-, tri-, tetra-) and different glycosides with likely aromatic groups (“glycoside-B_RI=”), hydrocarbon-linked groups (e.g., fatty acid, alkyl, or prenyl derivatives; “glycoside-C_RI=”), and glycosides resembling iridoid-type structures (“glycoside-T_RI=”) (Figure 1). Additionally, 16 amino acids and approximately 30 carboxylic acids were detected, including intermediates of glycolysis, fermentation, and the Krebs cycle. Lipid metabolism intermediates comprised seven free fatty acids (FFAs), two acylglycerols and nine sterols. There were seven terpenes and 17 phenolic compounds in the metabolic profiles.

To visualize the differences in metabolite content in the leaves of the studied species, a heatmap combined with hierarchical clustering was constructed (Figure 1). As shown, metabolites differed in their abundance. *Iris* species contained higher amounts of amino acids, organic acids, and alcohols compared to cinquefoils. The leaves of the hydrophytic *I. pseudacorus* accumulated significantly more sugars (sucrose, fructose, glucose, and others) in contrast to the other plants, including the mesophytic *I. sibirica*. Cinquefoils exhibited a significant tendency to accumulate pentoses and glycosides.

The diversity and median arbitrary accumulation of metabolites are visualized in the network diagram (Figure 2). Identified metabolites were clustered by structural similarity, while unidentified compounds were grouped based on mass spectral resemblance to annotated metabolites or to each other. Node sizes corresponded to median accumulation levels. Unsurprisingly, sucrose, fructose, glucose, and *myo*-inositol were found in high abundance. Among Krebs cycle intermediates, citrate, malate, and succinate exhibited relatively high accumulation, whereas 2-ketoglutarate, fumarate, and pyruvate were present at markedly lower levels. Amino acids were moderately abundant, with slightly higher levels of GABA and glutamate. Secondary metabolites such as quinate and catechin were notably accumulated too. The elevated levels of aromatic secondary compounds aligned with the observed high shikimate content. Ascorbate and its related metabolite, threonate, were also prominently accumulated. Importantly, a substantial proportion of metabolites remained unidentified, highlighting potential gaps in the current understanding of plant molecular diversity.

**Figure 3:**
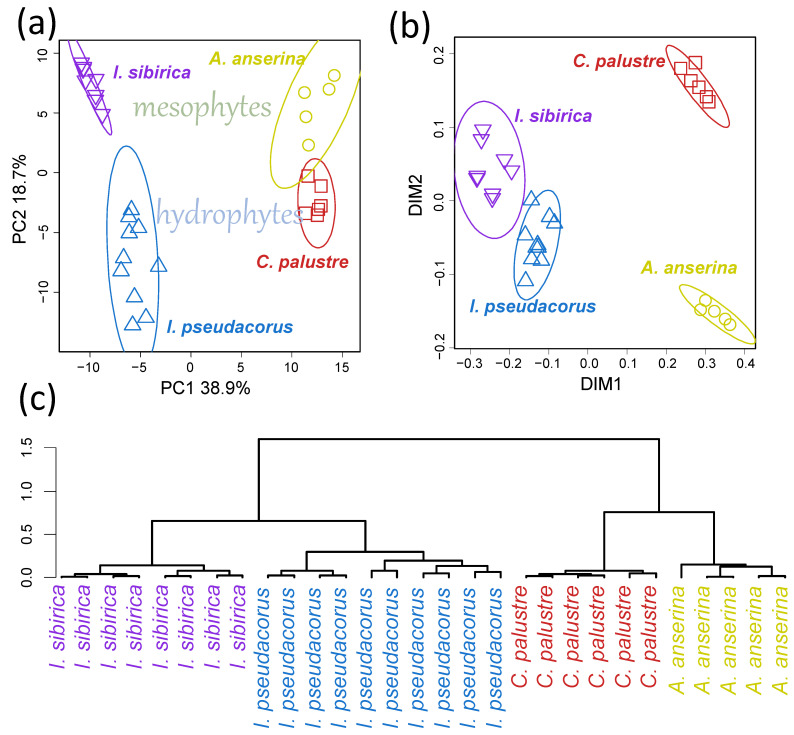
Unsupervised analysis of metabolite profiles. (**a**)—PCA score plot, (**b**)—Multidimensional scaling with Spearman’s distance (1-*r*), (**c**)—dendrogram of hierarchical clustering of metabolic profiles, Pearson’s distance (1-*r*), Ward method.

To identify general patterns in the similarities of metabolite profiles, we visualized them in a lower-dimensional space (Figure 3a) using Principal Component Analysis (PCA). Along PC1 (Principal Component 1), explaining 38.2% of the variance, representatives of the genus *Iris* were distinguished from those of the cinquefoils. PC2 (18.4%) and PC3 (14.6%) were associated with species-specific differences, with PC2 reflecting distinctions between hydrophytic and mesophytic species.

Using non-parametric values as similarity metrics reduces the impact of normalization on the results. We used Spearman’s rank correlation coefficient as a similarity metric for the representation of profiles in a low-dimensional space created using MDS (Multidimensional Scaling). On the MDS plot (Figure 3b), a clear separation between representatives of the genus *Iris* and the tribe Potentilleae was observed along the DIM1 (dimension 1) axis. Data likely indicated the primary gradient of variability associated with intergeneric differences. Species clustered according to ecological conditions (DIM2): hydrophytic and mesophytic species occupied distinct regions of the plot.

Hierarchical clustering of the metabolite profiles was also performed using Ward’s method with Pearson distance (Figure 3c). As shown, the metabolite profiles clustered distinctly according to species affiliation. All three unsupervised methods produced similar results, confirming the robustness of the findings and enabling us to conclude that there are significant metabolomic differences between the species investigated.

### 2.2. Intergeneric Metabolite Differences Between Irises and Cinquefoils

**Figure 4:**
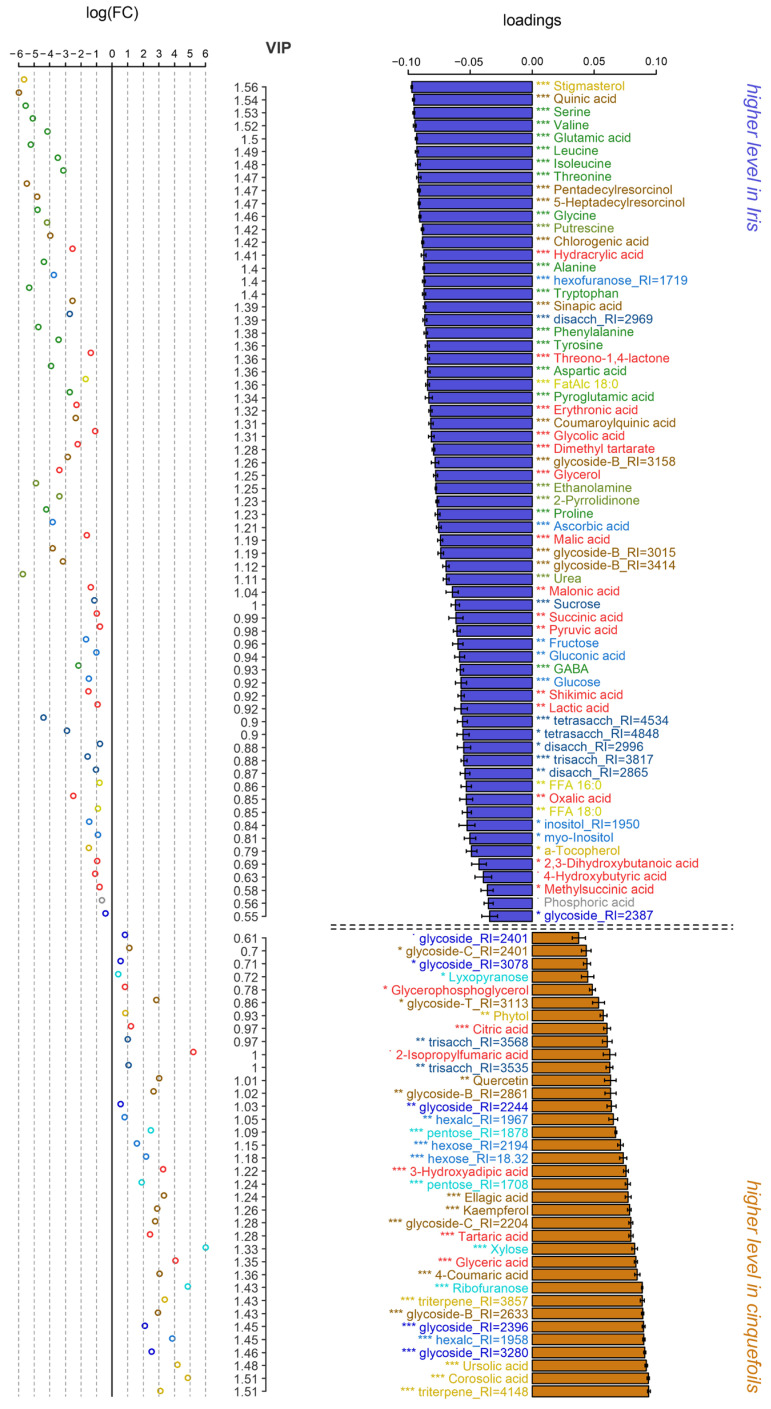
Differently accumulated metabolites in irises and cinquefoils. The bar plots on the right are factor loadings of predictive component from OPLS-DA model. Positive values correspond to higher level in cinquefoils. Colors mark chemical class in manner same as in Figure 1. Dot and asterisks—significance from Mann–Whitney–Wilcoxon tests: ˙ *p* < 0.1, * *p* < 0.05, ** *p* < 0.01, *** *p* < 0.001. Axis at the middle—VIPs from OPLS-DA. Scattering plot at the left is log_2_(FC), FC—fold changes: C_cinquefoils_/C*_Iris_*.

First, we identified differentially accumulating metabolites (DAMs) between *Iris* and cinquefoil plants. For this purpose, an OPLS-DA (Orthogonal Projections to Latent Structures-Discriminant Analysis) model was constructed, incorporating one predictive and one orthogonal component. The predictive component explained 37% of the variance, with Q^2^Y = 0.99 (*p* ≤ 0.001). DAMs were defined based on a VIP > 1 and the results of the Mann–Whitney–Wilcoxon test with Benjamini–Hochberg correction. As shown in Figure 4, *Iris* species exhibited higher levels of amino acids. Carboxylates displayed contrasting trends: while some showed a tendency toward greater abundance in *Iris*, cinquefoils accumulated more tartrate and glycerate. *Iris* species demonstrated higher levels of major Krebs cycle intermediates, such as malate and succinate, whereas cinquefoils exhibited elevated citrate levels. Notably, *Iris* also showed greater accumulation of pyruvate, lactate, glycolate and glycerol. Additionally, *Iris* species surpassed cinquefoils in the content of major sugars (fructose, glucose, and sucrose) while the latter were characterized by elevated pentose abundance.

Stigmasterol accumulated more prominently in *Iris*, whereas many triterpenoids were enriched in Potentilleae representatives. Phenolic metabolites contributed significantly to the differences between the groups. *Iris* leaves contained markedly higher levels of sinapic, quinic, chlorogenic, and coumaroylquinic acids, while cinquefoils exhibited greater amounts of kaempferol, quercetin, ellagic acid, coumaric acid, and glycosides with hydrocarbon groups.

### 2.3. Interspecific Metabolite Differences Among Irises Species

**Figure 5:**
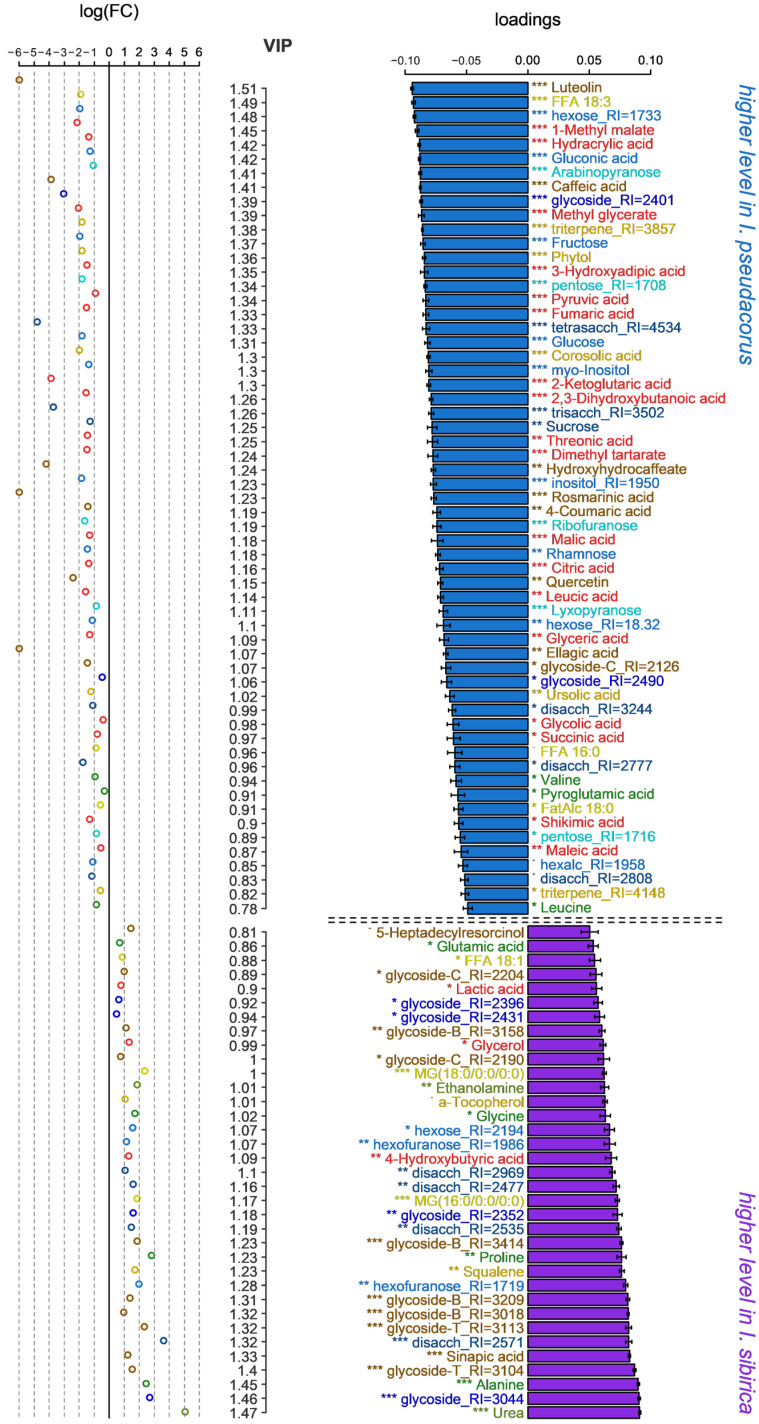
Differently accumulated metabolites in two *Iris* species. The bar plots on the right are factor loadings of predictive component from OPLS-DA model. Positive values correspond to higher level in *I. sibirica*. Colors mark chemical class in manner same as in Figure 1. Dot and asterisks—significance from Mann–Whitney–Wilcoxon tests: - *p* < 0.1, * *p* < 0.05, ** *p* < 0.01, *** *p* < 0.001. Axis at the middle—VIPs from OPLS-DA. Scattering plot at the left is log_2_(FC), FC—fold changes: C*_I.sibirica_*/C*_I.__pseudacorus_*.

The predictive component explained 38% of the variance, with Q^2^Y = 0.96 (*p* ≤ 0.001) in the OPLS-DA model comparing the two *Iris* species The hydrophytic *I. pseudacorus* accumulated the highest levels of all detected Krebs cycle intermediates, as well as pyruvate (Figure 5). Additionally, most other carboxylates were also more abundant in this species. Lactate was an exception, showing higher levels in *I. sibirica*. Interspecific differences were weakly associated with amino acid content. Notably, *I. pseudacorus* leaves exhibited greater amounts of Val, pyroglutamate, and Leu, while the opposite trend was observed for Glu, Gly, Pro, and Ala*. I. pseudacorus* also displayed higher levels of major sugars (sucrose, fructose, and glucose), *myo*-inositol, and gluconic acid. Differences in free fatty acids (FFAs) and triterpenoid content were also evident. Furthermore, the species diverged in their profiles of secondary metabolites.

### 2.4. Metabolite Differences Among Cinquefoil Species

**Figure 6:**
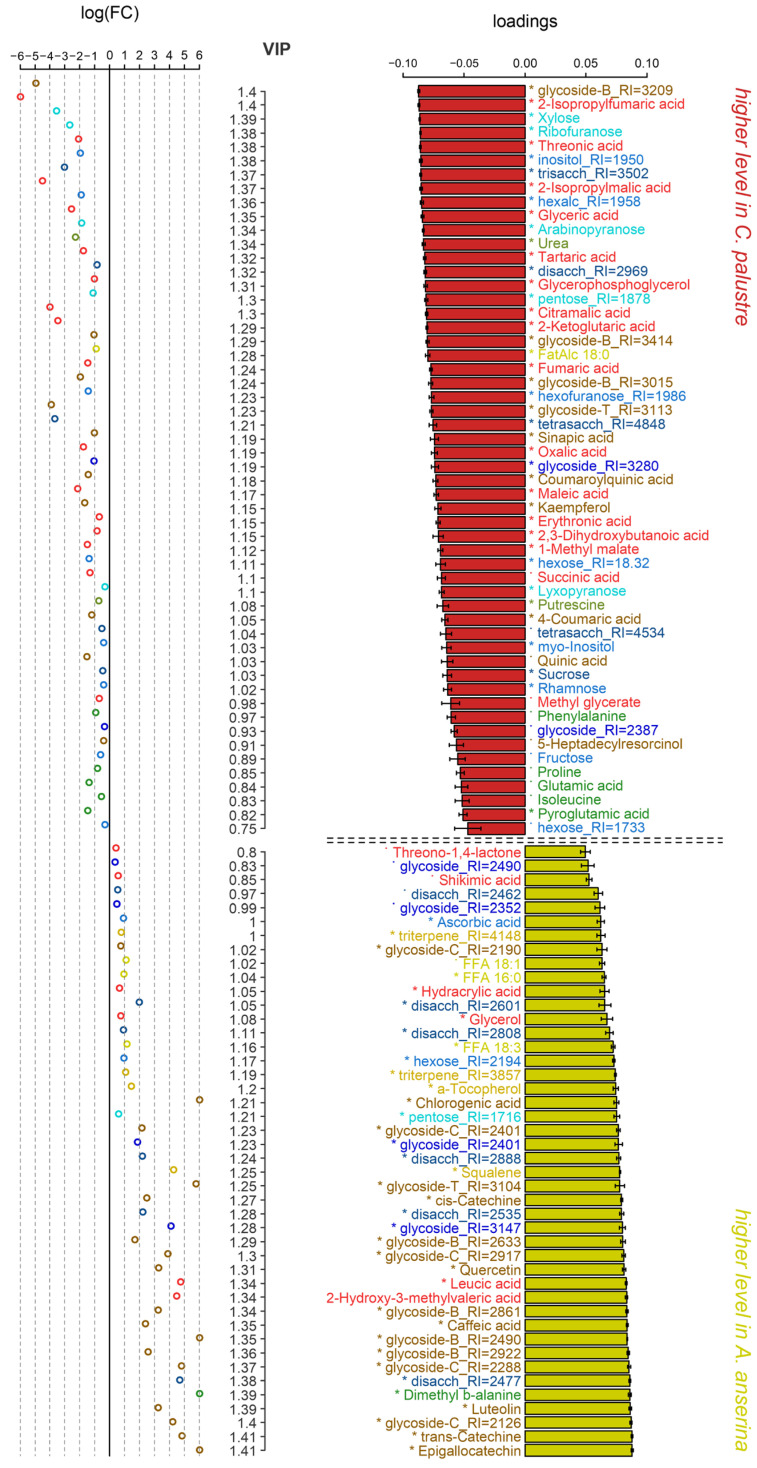
Differently accumulated metabolites in two cinquefoils. The bar plots on the right are factor loadings of predictive component from OPLS-DA model. Positive values correspond to higher level in *A. anserina*. Colors mark chemical class in manner same as in Figure 1. Dot and asterisks—significance from Mann–Whitney–Wilcoxon tests: ˙ *p* < 0.1, * *p* < 0.05. Axis at the middle—VIPs from OPLS-DA. Scattering plot at the left is log_2_(FC), FC—fold changes: C*_A. __anserina_*/C*_C.__ palustris_*.

In the OPLS-DA model comparing *C. palustre* and *A. anserina*, the predictive component explained 47% of the variance, with Q^2^Y = 0.98 (*p* = 0.003). The hydrophytic *C. palustre* accumulated significantly higher levels of Krebs cycle intermediates and several other carboxylates (Figure 6). Amino acids contributed minimally to interspecific differences. Among nitrogen-containing compounds, *C. palustre* exhibited greater accumulation of pyroglutamate, putrescine and urea. Sugars, particularly sucrose, were more abundant in *C. palustre* too. Notably, *A. anserina* displayed a larger ascorbate pool. Lipophilic compounds showed weak association with species-specific variation; however, *A. anserina* demonstrated elevated levels of free fatty acids (FFAs) and triterpenes, including squalene. As expected, the taxonomic groups differed markedly in their secondary metabolite profiles. Specifically, *A. anserina* possessed higher reserves of catechins and other phenolic compounds.

### 2.5. Hydrophytic Metabolic Traits

**Figure 7:**
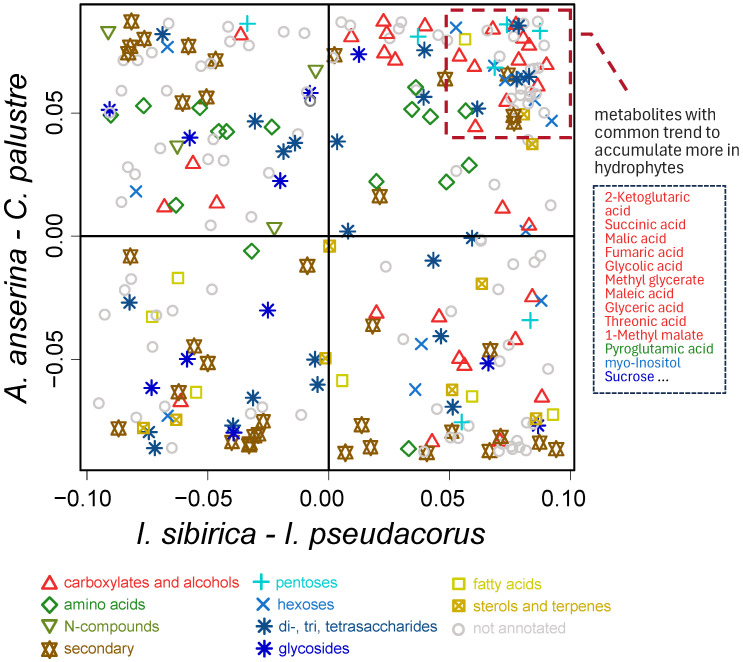
Comparative analysis of DAMs in hydrophytes and mesophytes. SUS (shared and unique structures) plot—ordination of metabolites in the space of the loadings from two OPLS-DA models.

In each of the two species pairs, one is a hydrophyte and the other a mesophyte. We attempted to identify common metabolic traits of the hydrophytic species. To achieve this, we first constructed an SUS-plot, which displays the distribution of metabolites in the loading space of the predictive components from OPLS-DA models used for species classification. Metabolites accumulating in higher concentrations in the hydrophytic *C. palustre* and *I. pseudacorus* were clustered in the upper right quadrant (Figure 7). Overall, hydrophytic plants exhibited a tendency to accumulate organic and amino acids. Hydrophytic adaptation was associated with elevated levels of Krebs cycle intermediates and several other carboxylates. Notably, hydrophytes also accumulated higher amounts of sucrose and *myo*-inositol. Among amino acids, pyroglutamate showed the most pronounced accumulation in hydrophytic species.

## 3. Discussion

Leveraging the broad ecological plasticity of the studied taxa, our comparative study examined both mesophytic and hydrophytic species to elucidate how their distinct ecological niches influence their metabolic processes and the accumulation of secondary metabolites. Specifically, we compared typically mesophytic species such as *I. sibirica* and *A. anserina* with classic hydrophytes like *I. pseudacorus* and *C. palustre*. All samples for metabolite profiles analysis were collected from native environments and were not treated by external flooding, which represents the result of continuous adaptation to different habitats.

Metabolomic studies of representatives of genus *Iris* and the tribe Potentilleae that are available in the literature are exclusively devoted to the elucidation of their bioactive compounds for guaranteed use in pharmacology and therapy. Comparative profiling of rhizomes and aerial parts of *I. pseudacorus* plants from Egypt and Japan was carried out using the UPLC-ESI-MS/MS method [32]. A total of 43 compounds were identified, such as flavonoids (quercetin, isorhamnetin), isoflavonoids (tectogenin, irilin D), phenolic acids (galloyl glucose) и xanthones. Rhizome extracts have demonstrated high antioxidant activity [32]. Metabolomes of three *Iris* species were studied (*I. confusa, I. pseudacorus* и *I. germanica*) for taxonomic differentiation [31]. Metabolic profile revealed key compounds that distinguish *I. pseudacorus* from other *Iris* species. The underground parts were found to accumulate elevated levels of riboflavin, glutamine, and allantoic acid. In turn, *I. confusa* was characterized by high sucrose content, while *I. germanica* contained more citric acid, glucose, and mannitol. The leaves of *I. pseudacorus* are characterized by high levels of organic acids (fumaric, isocitric, pyruvic), sugars (fructose, raffinose) and sugar alcohols (mannitol), as well as vitamins (riboflavin, ascorbic acid) [31]. Our GC-MS profiling detected an elevated abundance of carboxylates (fumarate, succinate, citrate, pyruvate), sugars (glucose, fructose, rhamnose and sucrose), phenolic acids and flavonoids (Figure 4 and Figure 5). The phytochemical composition of *I. sibirica* has been poorly studied. HPLC-MS/MS analysis of *I. sibirica* leaves revealed 36 compounds, mainly isoflavones and their glycosides [33]. In our case *I. sibirica* leaves accumulated greater amounts of glycosides (Figure 5). Both species contained high levels of antioxidants: ascorbate, α-tocopherol, and squalene.

Early reported GC-MS analysis of *C. palustre* revealed elevated levels of caffeic, erythronic, galactaric acids, *myo*-inositol and monosaccharides (arabinose, isomaltose, fucose) in the above-ground part, while the underground part was characterized by the dominance of glucose, ribose and xylose [34]. We found high abundance of *myo*-inositol, pentoses (arabinose, ribose, xylose), fructose and sucrose, as well as organic and amino acids in *C. palustre* leaves (Figure 6). The caffeate level was greater in *A. anserina* extracts.

In a previous GC-MS study of *A. anserina*, 53 compounds were detected, including organic acids (citric, malic), sugars (arabinose, xylose, glucose), sugar alcohols (*myo*-inositol, mannitol) and amino acids (Ala, Pro) [29]. Of these, 36 were confirmed by standards. HPLC-MS/MS analysis revealed 17 secondary metabolites, including chlorogenic acid, quercetin-3-O-glucoside and genistein (first detected in this species) [29]. The HPLC-MS/MS method was used to further study the pharmacokinetics of rosamultin, a key triterpenoid saponin of *A. anserina* roots, confirming its bioavailability [30]. Our profiling identified sugars, polyols, organic and amino acids in the leaves of silverweed, but their abundances were less than that of *C. palustre* (Figure 6). On the contrary, levels of caffeate, chlorogenate, quercetin and luteolin as well as diverse glycosides were elevated. *A. anserina* leaves accumulated high amounts of lipophilic compounds including triterpenoids.

This GC-MS study revealed significant differences in the metabolic profiles of the leaves of the mesophytes *I. sibirica* and *A. anserina* compared to the hydrophytes *I. pseudacorus* and *C. palustre* (Figure 1 and Figure 3). The diversity and relative accumulation of metabolites reflect the adaptation of plants to different environmental conditions. Among the intermediates of the tricarboxylic acid cycle, succinate and fumarate accumulated in hydrophytes, which is likely due to the suppression of succinate dehydrogenase and the activation of the reverse pathway of the dicarboxylic acid part of the cycle [3]; the electron transport chain is also suppressed, which leads to branching of the Krebs cycle and reversal of dicarboxylic part [3,14,15]. This may cause the observed accumulation of succinate and fumarate, but a decrease in 2-ketoglutarate was due to the blocking of oxidative reactions. For example, in rice (*Oryza sativa*), after 6 days of anoxia [35] or 3–7 days of submergence [19] a decrease in the 2-ketoglutarate content was observed, despite an increase in other intermediate products of the Krebs cycle (succinate, fumarate, citrate). In contrast, in eelgrass (*Zostera marina*), an increase in the 2-ketoglutarate content was revealed in leaves under anoxia [23]. In our case the content of 2-ketoglutarate in both species of hydrophytes was also higher than in mesophytes (Figure 1 and Figure 7). The revealed accumulation of glycolytic metabolites (pyruvate, glycerate) was noted in *I. pseudacorus* (Figure 5), which is consistent with the dynamics of these compounds in hydrophytic *Ranunculus* and *Epilobium* species, which we identified previously [24,25].

The amino acid profile varied significantly among *Iris* and cinquefoils, with increased accumulation of all identified amino acids in *Iris* species (including the phosphoglycerate family (Ser and Gly), the shikimate family (Phe, Tyr, and Trp), the pyruvate family (Ala, Leu, and Vale), and the Asp family (Thr and Ile), as well as the Glu family (Glu, Pro, and GABA)). The accumulation of amino acids from these families is part of the “metabolic signature” of oxygen deficiency [2]. This is associated with the restructuring of primary metabolism to maintain energy balance, provide nitrogen assimilation, synthesis of nitrogenous compounds, osmotic and pH adjustment as well as the alternative NAD(P)H reoxidation pathways avoiding cytosolic lactoacidosis and toxicity [2,3,9,36]. However, accumulation of some amino acids from this list (Ala, Gly, and Pro) was found not in the hydrophytic *I. pseudacorus* but in mesophytic *I. sibirica* (Figure 1 and Figure 5). In cinquefoils, a higher Pro abundance was shown in the hydrophytic *C. palustre*, unlike *A. anserina* (Figure 1 and Figure 6). Pro is an imino acid and its content increases under the influence of stressors. It plays a key role in osmotic adjustment, promoting water retention in cells and protecting macromolecules from destruction under various stress conditions (e.g., drought and salinity) [37,38,39]. Pro also functions as an antioxidant [39]. Hydrophytic *I. pseudacorus* tended to accumulate shikimate, unlike mesophytic *I. sibirica* (Figure 1 and Figure 5). However, no significant increase in the shikimate family of amino acids was recorded in the hydrophytic iris, while *C. palustre* had higher Phe content. Nevertheless, the amount of phenolic compounds in the leaves of *I. pseudacorus* was increased (Figure 5). There is extensive literature data on the accumulation of shikimate family amino acids (Phe, Tyr, and Trp) in plants under hypoxia/anoxia among oxygen deficiency-tolerant species, for example, rice [35], hydrophytic *Epilobium hirsutum and E. palustre* [24], and *Ranunculus sceleratus* [25]. Phenylalanine is an intermediate product in the synthesis of phenolic compounds such as flavonoids, lignins, and tannins [40].

In hydrophytic species, in contrast to mesophytic ones, a pronounced accumulation of glucose, fructose, and sucrose was observed (Figure 1, Figure 5 and Figure 6), which is consistent with their increased tolerance to hypoxia. These soluble sugars serve as substrates for glycolysis, providing ATP synthesis under conditions of oxygen deficiency [3,9]. Glycolysis requires a sufficient amount of carbohydrates due to its high consumption of sugars [3]. Tolerant plants of aquatic and semi-aquatic habitats accumulate significant amounts of carbohydrates in their rhizomes—up to 50% of their dry weight [9,41,42]. Proper carbohydrate mobilization can enhance plant survival under oxygen-deficient conditions. Soluble sugars are present in plants in limited quantities and are quickly consumed during anaerobic fermentation. For example, *Acorus calamus*, with large starch reserves in its rhizomes, is able to maintain a low metabolic rate even when completely flooded, thus surviving for long periods [9].

Ascorbate accumulated in the hydrophyte *I. pseudacorus* (Figure 1). However, threonate, a derivative of the four-carbon monosaccharide threose, was also observed in both hydrophytes. Threonate can be formed both from threose and by ascorbate degradation [43]. It is also worth noting that threonic acid accumulation is a common marker of hydrophytic metabolism (Figure 1, Figure 5 and Figure 6). This compound was significantly elevated in hydrophytic species of *Epilobium* and *Ranunculus* compared to mesophytic representatives [24,25]. Ascorbate has been described as a key component of the antioxidant system, neutralizing reactive oxygen species (ROS), which are actively formed during the restoration of aerobic conditions after hypoxia [2]. Moreover, in a flood-tolerant plant (rice), the ascorbate-glutathione cycle demonstrated more stable operation under conditions of oxygen deficiency and reoxygenation [44]. Among the cinquefoils, conversely, ascorbate accumulated more in the mesophytic *A. anserina* along with other antioxidants—α-tocopherol and squalene.

Among the secondary metabolites, hydrophytes from both groups showed a slight tendency to accumulate coumaric acid (Figure 1, Figure 5 and Figure 6), a key intermediate in the synthesis of flavonoids and lignins [40]. The hydrophyte *I. pseudacorus* differed from the mesophyte *I. sibirica* in the accumulation of caffeate, rosmarinate, coumarate, ellagate, quercetin, and luteolin. In turn, *I. sibirica* contained more sinapic acid (Figure 5). Luteolin and quercetin are flavonoids with antioxidant activity. The accumulation of quercetin is also characteristic of other hydrophytes, for example, *E. hirsutum* and *E. palustre* [24]. Hydrophytic willow-herbs showed the same tendency for this metabolite content when grown in a swampy environment. A protective effect against hypoxia and subsequent reaeration was demonstrated in *T. aestivum* cultivar “Orenburgskaya 22” seedlings pretreated with quercetin [45]. Quercetin—an antioxidant—likely reduces the formation of reactive oxygen species (ROS) [45]. However, quercetin accumulated less in hydrophytic *C. palustre* than in mesophytic *A. anserina* (Figure 6). The antioxidant properties of luteolin are primarily studied in medical research as a pharmacological agent, rather than in the context of its effects on plants [46]. Ellagic acid is a polyphenol, a component of tannins, and an antioxidant studied in animal cells. Ellagic acid inhibits lipid peroxidation in rat liver microsomes [47]. Catechins have antioxidant and antimicrobial properties and may also play a role in plant defense against phytophages [48], but their role in plant adaptation to hypoxia needs to be studied.

In the hydrophyte *C. palustre*, we found and identified fewer secondary compounds than in the mesophyte *A. anserina*. The hydrophytic cinquefoil accumulated kaempferol, quinic, coumaric and sinapic acid. Kaempferol, along with quercetin and luteolin, is a flavonoid. Some plants exhibit hypoxia tolerance primarily due to flavonoid synthesis. Using two tobacco varieties (tolerant and non-tolerant to the combined effects of hypoxia and cold), it was demonstrated that the tolerance of the Honghua variety was due to the accumulation of higher amounts of flavonoids, unlike that of the K326 variety. Furthermore, exogenous treatment with anthocyanidin, flavones, and quercetin increased the viability of the non-tolerant variety [49]. One of the mechanisms of the antioxidant action of flavonoids is the activation of antioxidant defenses (e.g., upregulation of antioxidant enzymes with ROS scavenging capacity) [50]. Previous studies have shown that increased flavonol accumulation reduces ROS levels and promotes antioxidant enzyme activity under drought stress [51,52]. Hypoxia-tolerant plants have been shown to exhibit increased peroxidase activity under the influence of anoxia and reaeration [53].

The hydrophyte *I. pseudacorus* differed from the mesophyte *I. sibirica* in its content of lipids and their derivatives. The hydrophyte accumulated FFA 18:3 (linolenic acid), FFA 16:0 (palmitic acid), phytol, and triterpenes. Under abiotic stress, plants typically exhibit a decrease in chlorophyll content, stimulation of lipid catabolism and an increase in cell membrane permeability [54], which may explain the small accumulation of phytol and fatty acids in the hypoxia-tolerant species. Under conditions of oxygen deficiency, lipid degradation and changes in their structure occur, especially in hypoxia-sensitive plants [3]. Among cinquefoils the accumulation of fatty acids and terpenes was higher in the mesophyte *A. anserina*.

In our previous studies, when comparing the metabolomes of hydrophytes with mesophytes, we found a tendency towards accumulation of organic, fatty and proteinogenic amino acids (as well as GABA) in hydrophytic species of willowherbs (*E. hirsutum*, *E. palustre*) [24] and buttercups (*R. sceleratus*) [25]. In general, the same tendency is observed in the hydrophytic *I. pseudacorus* in comparison with the mesophytic *I. sibirica*, although amino acids prevailed in mesophyte (Figure 5). Among cinquefoils, the dynamics of alteration of primary metabolism associated with the ecological niche were revealed to be not that clear, except for the accumulation of some organic and amino acids in the hydrophytic *C. palustre* (Figure 6). Nonetheless, common hydrophytic metabolic traits were shown in both, *I. pseudacorus* and *C. palustre*: there was an increase in abundance of carboxylates (succinate, fumarate, 2-ketoglutarate, etc.), some amino acids and sugars (Figure 7). Mesophytes were characterized by greater content of glycosides. Differences between the two species of the tribe Potentilleae significantly depended on the accumulation of secondary compounds. *A. anserina* growing at normoxic conditions accumulated more flavonoids with antioxidant activity (quercetin, luteolin, catechins) and lipids than the hydrophyte *C. palustre*. Although under hypoxia, the need for antioxidants should theoretically be higher due to the activation of lipid peroxidation and the generation of ROS and RNS [3,5,6,7]. Probably, in hydrophytic *C. palustre* plants, other compounds (e.g., glutathione) are more involved in neutralizing oxidative processes. Thus, in flood-tolerant rice, it is the coordinated functioning of the ascorbate-glutathione cycle that provides effective antioxidant protection [44]. This may also be due to the possible influence of other abiotic factors —primarily light exposure on *A. anserina*—since the plants were collected in their natural habitat, or to individual characteristics of the species.

Summing up the comparison of species adapted to relatively dry or wet ecological niches showed alteration in several groups of metabolites. Mesophytes were mostly characterized with the accumulation of glycosides, predominantly of phenolic nature. Such an adaptation probably reflects the necessity of higher antioxidant status or photoprotection. While hydrophytes manifested the increase in sugars, organic acids and number of lipids which could be a sign of a metabolic shift in direction to anaerobic processes. It is important to admit that such metabolic signature was obtained in plants without experimental flooding at normal growing conditions which persisted over a long period of time in dryland (for mesophytes) or wetland (for hydrophytes).

## 4. Materials and Methods

### 4.1. Plant Material

The subjects of this study are representatives of the genus *Iris* Tourn. ex L. (irises), which are extensively distributed and widely cultivated in the temperate and subtropical zones of the Northern Hemisphere, as well as representatives of cinquefoils (or silverweeds, tribe Potentillinae Sweet), *Argentina anserina* (L.) Rydb. and *Comarum palustre* L.—two species which are widely distributed in the temperate and boreal zones of the Holarctic.

*Iris* is the largest genus within the family Iridaceae Juss., comprising approximately 330–355 species according to current estimates [55,56], with the greatest diversity found in the Mediterranean region, Southwestern, and Eastern Asia. Although species within this genus exhibit significant variability in their ecological preferences, most tend to favor dry, semi-desert, or rocky mountainous habitats [26]. There is no single consensus on the systematics within the genus; however, most researchers refer to the classification systems of G.I. Rodionenko [57] or B. Mathew [58], which divided the genus *Iris* into several subgenera further subdivided into 12 sections. The broad ecological plasticity of the studied genus allows for the comparison of typical mesophytic iris (*I. sibirica* L.) with classic hydrophyte (*I. pseudacorus* L.) [59], and also allows these ecological differences to be reflected in the characteristics of metabolism and the accumulation of various secondary metabolites.

*A. anserina* (L.) Rydb. (basionym *Potentilla anserina* L., common silverweed or silver cinquefoil) is a species of genus *Argentina* Hill within the tribe Potentilleae Sweet, which belongs to the Rosaceae Juss. family. This genus is known for its extensive distribution and includes approximately 70 species [60,61]. *A. anserina* is found in Europe, Northern Asia, and North America, preferring moist and sandy soils, frequently in wet meadows, along water bodies, and in boggy areas. The species has broad ecological amplitude, favoring moist, sandy, or silty soils, but can adapt to various soil types provided they are well-drained [62].

*C. palustre* L. (marsh cinquefoil) belongs to the genus *Comarum* L., which is also part of the Rosaceae family. This monotypic genus is sometimes included in *Potentilla* L. by some taxonomists due to its close taxonomic relationship. Marsh cinquefoil is found in the northern parts of Europe, Asia, and North America, typically inhabiting wetlands, peat bogs, and the edges of water bodies. The plant is adapted to cold and wet conditions and usually grows in boggy or other waterlogged environments [63].

Specimens were collected from the “Sergievka” park and its surrounding area (Petrodvorets district of St. Petersburg). Collection site coordinates for *I. pseudacorus* are 59.9005320 N, 29.8389347 E at a marshy reed swamp along the shore of the Gulf of Finland, and for *I. sibirica:* Botanical garden of St. Petersburg State University, 59.941934 N, 30.295583 E. Collection coordinates for *A. anserina*: 59.8845045 N, 29.8272226 E, *C. palustre*: 59.9000937 N, 29.8423207 E. The “Sergievka” park is located near the Gulf of Finland, which imparts maritime characteristics to its climate. The primary climatic factor is the intense movement of air masses, leading to frequent precipitation even during the winter months. February is the coldest month, with an average daily temperature dropping to −8.1 °C. In summer, the influence of western winds intensifies, resulting in significant cloudiness and abundant precipitation. July is the hottest month, with an average daily temperature reaching plus 16–17 °C [64]. Plants were collected at midday on 17 July 2017 (18.5 °C, atmospheric pressure 1007.9 hPa, relative air humidity 71%, south-western wind, 4 m/s, irradiance 600 µmol·m^−2^·s^−1^) and 17 July 2018 (18 °C, atmospheric pressure 1009.3 hPa, relative air humidity 76%, eastern wind, 5 m/s, irradiance 550 µmol·m^−2^·s^−1^). Samples collected at different years were clustered together.

### 4.2. Sample Preparation

Leaves of hydrophytic species (*I. pseudacorus*, *C. palustre*) were gathered from plants growing in water or in its vicinity, while leaves of mesophytic species (*I. sibirica*, *A. anserina*) were obtained from plants growing on soils with normal moisture and aeration. Leaves were taken from healthy plants, undamaged by pathogens and phytophages, and of the same generation (3rd from the apex) and approximately the same size. Then, they were weighed to 200 mg using a portable electronic scale, cut into small segments, and fixed in 1 mL of methanol. Samples were collected in mid-July. We analyzed 5–8 biological replicates collected from different plants. Upon delivery to the laboratory, the plant material was immediately subjected to extraction and evaporation as described earlier [24,25]. The dried extracts were stored under a nitrogen atmosphere at −80 °C until further analysis.

For chromatographic analysis, the dried extracts were dissolved in 100 µL of pyridine each containing the internal standard C23 (tricosane, Sigma-Aldrich, St. Louis, MO, USA) and derivatized by adding of 100 µL of the silylating agent (1% solution of trimethylchlorosilane in bis(trimethylsilyl)-N, O-trifluoroacetamide, Sigma-Aldrich, St. Louis, MO, USA) [24,25].

To test the analytical procedures, quality control (QC) samples of a mixture of extracts from the leaves of fifteen vascular plant species were used (Figure S1).

### 4.3. Gas Chromatography-Mass Spectrometry (GC-MS)

GC-MS was carried out as described earlier [65].The analysis was conducted using an Agilent 6850 gas chromatograph coupled with mass-selective quadrupole detector Agilent 5975, under control of MassHunter software (v. 10.1, Agilent Technologies, Santa Clara, CA, USA). Sample injection was carried out in splitless mode with a volume of 0.5 µL per analysis. Chromatographic separation was achieved using a DB-5ms capillary column (Agilent Technologies, Santa Clara, CA, USA). Ultra-pure helium served as the carrier gas at a constant flow rate of 1 mL/min, with the injector maintained at 250 °C. The oven temperature program began at 70 °C with a 6 °C/min ramp until reaching 320 °C. Detection was operating in full-scan mode across a mass-to-charge (*m*/*z*) range of 50–700. The ion source and quadrupole analyzer temperatures were maintained at 230 °C and 150 °C, respectively. All experiments were performed using the instrumentation at St. Petersburg State University’s Resource Center for Molecular and Cellular Technologies (State Assignment no. 125022803066-3).

### 4.4. GC-MS Data Analysis

Chromatographic data analysis was performed using PARADISE software (v. 6.0.1, Department of Food Science Faculty of Science, University of Copenhagen, Denmark, ref. [66] in conjunction with the NIST MS Search 2.4 platform (National Institute of Standards and Technology (NIST), Gaithersburg, MD, USA). Metabolite detection and spectral deconvolution were automated using AMDIS (v. 2.71, Automated Mass Spectral Deconvolution and Identification System, NIST). Compound identification integrated mass spectral data with Kovacs retention indices. Retention index calibration was achieved through analysis of a saturated *n*-alkane series under identical chromatographic conditions. For identification we used three major databases: the NIST20 spectral library, the Golm Metabolome Database (GMD, Potsdam, Germany) [67], and a specialized phytochemical repository maintained by the Laboratory of Analytical Phytochemistry at the Komarov Botanical Institute, RAS (St. Petersburg, Russia; State Assignment no. 124020100140-7).

### 4.5. Statistical Processing of Metabolomic Data

Metabolomic data processing was conducted in R (v 4.3.1) [68]. Raw data were normalized to the sample median. Outliers were identified and removed using the Dixon test (R package outliers v 0.15) [69]. Subsequently, the data were log-transformed and scaled to unit variance. Metabolites absent in specific samples but detected in technical replicates were considered missing values due to technical artifacts and were imputed via the k-nearest neighbors (KNN) algorithm using the impute package (v. 1.80.0) [70]. Multivariate statistical analysis included Principal Component Analysis (PCA), performed with pcaMethods (v. 1.98.0) [71], and Orthogonal Projections to Latent Structures-Discriminant Analysis (OPLS-DA), implemented in the ropls package (v. 1.32.0) [72]. The predictive component loadings and Variable Importance in Projection (VIP) scores were used to identify metabolites that were significantly associated with the factors of interest. Model quality was assessed using the R^2^Y (goodness-of-fit) and Q^2^Y (goodness-of-prediction) metrics, with statistical significance validated by permutation testing (*p* < 0.01). Visualization of metabolite abundance patterns was achieved by constructing heatmaps with the ComplexHeatmap circlize package (v. 0.4.16) [73]. Graph was constructed using Cytoscape software (v. 3.10.2) [74].

## 5. Conclusions

Thus, significant differences were identified in the leaf metabolite profiles of mesophytes and hydrophytes from the genus *Iris* and cinquefoil species. The metabolome of hydrophytes *I. pseudacorus* and *C. palustre* was characterized by the accumulation of Krebs cycle dicarboxylic acids, unlike that of mesophytes *I. sibirica* and *A. anserina*. Hydrophytes also exhibited an accumulation of some amino acids. Similar changes in the metabolic profile are characteristic of other hypoxia-tolerant plants. These results contribute to the identification of “metabolic fingerprints” specific to tolerant plants under oxygen-deficient conditions and highlight the complexity of adaptations to hypoxia, including the restructuring of primary metabolism and species specificity. Mesophytes were characterized by the accumulation of glycosides, predominantly of phenolic nature. Intraspecific differences were mostly associated with secondary compounds. This requires further study of the molecular mechanisms underlying species environmental tolerance and chemotaxonomy.

Understanding plant metabolic adaptation to various conditions of oxygen availability as explored in this study can be used for metabolic phenotyping (chemotyping) in the assessment of plant tolerance to oxygen deprivation for sustainable agriculture, ecosystem management, and biotechnological solutions.

## Data Availability

The original contributions presented in this study are included in the article/Supplementary Material. Further inquiries can be directed to the corresponding author.

## Supplementary Materials

The following supporting information can be downloaded at: https://www.mdpi.com/article/10.3390/ijms27041814/s1.

## Abbreviations

The following abbreviations are used in this manuscript:

DAM	Differentially Accumulating Metabolite
DIM	DIMension
FFA	Free Fatty Acid
GABA	γ-AminoButyric Acid
KNN	k-nearest neighbors
LOES	Low Oxygen Escape Syndrome
LOQS	Low Oxygen Quiescence Syndrome
MDS	Multidimensional Scaling
OPLS-DA	Orthogonal Projections to Latent Structures-Discriminant Analysis
PC	Principal Component
PCA	Principal Component Analysis
QC	Quality Control
RNS	Reactive Nitrogen Species
ROS	Reactive Oxygen Species
SUS	Shared and Unique Structures
VIP	Variable Importance in the Projection
