# Supplementary material for: Metabolite Profiling of Leaves of Irises and Cinquefoils of Hydrophytic and Mesophytic Nature
Source: Int J Mol Sci. 2026 Feb 13;27(4):1814. doi: 10.3390/ijms27041814 (PMC12940778; doi:10.3390/ijms27041814)
Supplement: Supplementary file 1 [file ijms-27-01814-s001.zip › ijms-4093314-Figure S1.pdf]

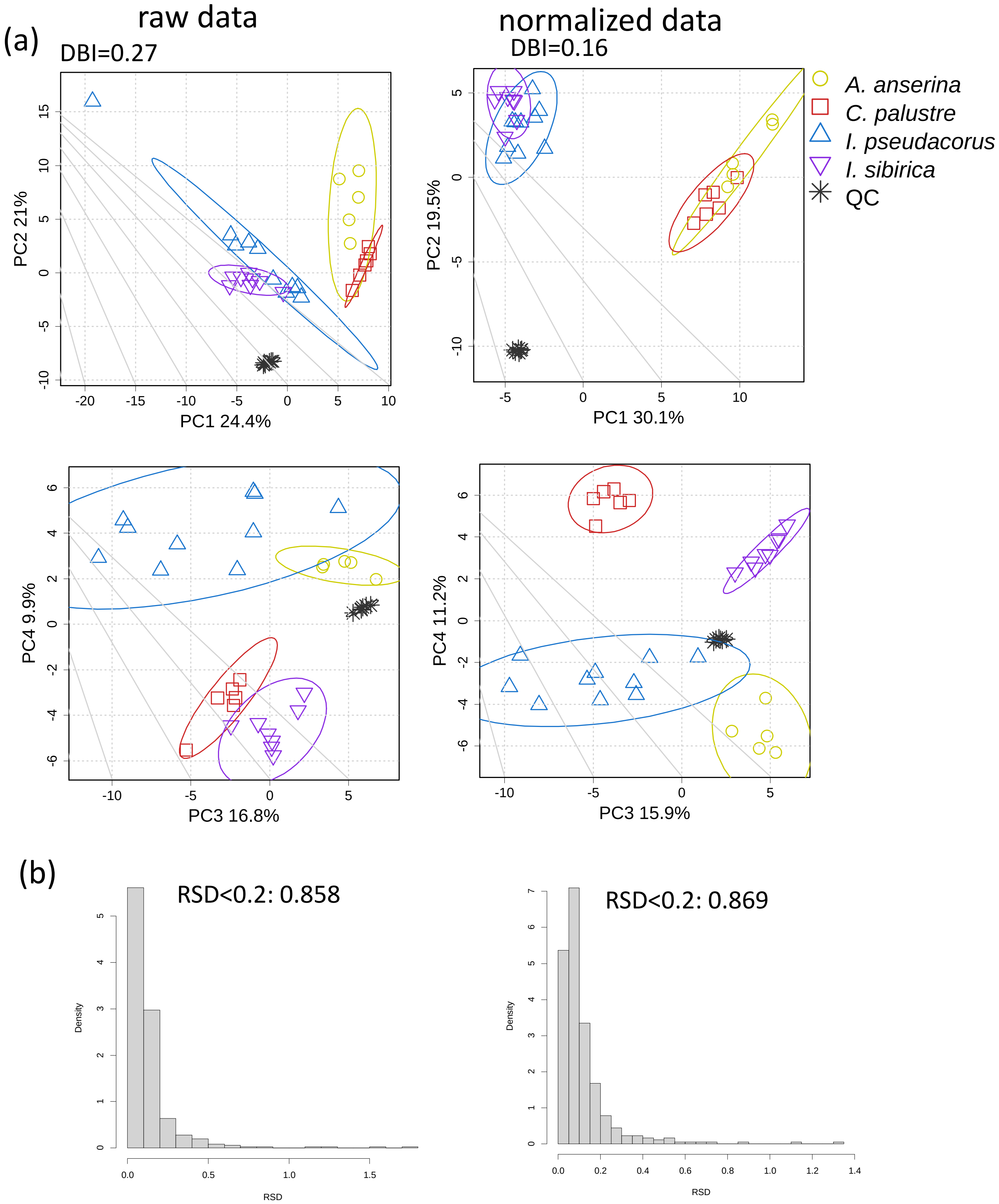

Figure S1. Normalization and QC diagnostics. (a) PCA score plots of profiles from 4 species and QC samples (mix of extracts from 15 species of vascular plants). DBI - Davies–Bouldin index. **(b)** Histograms of RSD (relative standard deviation) from QC samples (raw at the left and normalized per sample median at the right). Proportions of RSD < 0.2 are given.

For quality control (QC) the extracts from 15 species of vascular plants (*Argentina anserina*, *Comarum palustre*, *Iris pseudacorus*, *I. sibirica*, *Epilobium angustifolium*, *E. palustre*, *Filipendula ulmaria*, *Lemna minor*, *Myosotis arvensis*, *M. palustris*, *Oryza sativa*, *Ranunculus sceleratus*, *Rorippa palustris*, *Rumex aquaticus*, *Stachys palustris*) obtained as described in 4.2. section were mixed, divided in 15 equal parts and dried. Further manipulations were done as mentioned in 4.2-4.5 sections of Materials and Method.
